# Comparison of Fat Harvested from Flank and Falciform Regions for Stem Cell Therapy in Dogs

**DOI:** 10.3390/vetsci8020019

**Published:** 2021-01-25

**Authors:** Alexandra Jifcovici, Miguel A. Solano, Noel Fitzpatrick, Laurent Findji, Gordon Blunn, Anita Sanghani-Kerai

**Affiliations:** 1 Fitzpatrick Referrals, Surrey GU7 2QQ, UK; miguels@fitzpatrickreferrals.co.uk (M.A.S.); noelf@fitzpatrickreferrals.co.uk (N.F.); laurentf@fitzpatrickreferrals.co.uk (L.F.); anitas@fitzpatrickreferrals.co.uk (A.S.-K.); 2 School of Pharmacy and Biomedical Sciences, University of Portsmouth, Portsmouth PO1 2DT, UK; gordon.blunn@port.ac.uk

**Keywords:** stem cells, degenerative, joint, canine, weight, age, treatment, laboratory

## Abstract

Background: Adipose tissue has recently gained attention as a source of mesenchymal stem cells (AdMSCs) for applications in treating degenerative joint disease in veterinary patients. This study aimed to quantify the stromal vascular fractions (SVFs) and colony forming units (CFU) of AdMSCs from the falciform and flank regions and compare dogs of different ages and weights. Methods: Fat tissue was harvested from the flank (21 dogs) and falciform regions (17 dogs). The fat tissue was enzymatically digested and the number of nucleated cells in the SVF was counted. The SVF was cultured in vitro and the cell growth was assessed by counting the CFU per gram of fat and the aspect ratio of the cells. Conclusions: There was no significant difference in the number of nucleated cells in the SVF from the two sites. The CFU/g of fat from falciform was 378.9 ± 293 g and from flank was 486.8 ± 517 g, and this was also insignificant. Neither age nor weight of the patient had an impact on the SVF or CFU/g. No surgical complications were reported from either of the sites. Harvesting fat for stem cell therapy for intra-articular therapy of degenerative joint disease can be an easy and fast process when obtaining the fat either from the flank or the falciform region, and it is not age or weight dependent. The harvest site for clinical canine patients can be left to the surgeon’s discretion and comfort.

## 1. Introduction

Intra-articular therapy using mesenchymal stem cells (MSCs) is a recent therapeutic approach for the treatment of degenerative joint disease in dogs. The effect of MSCs involves interactions with the host microenvironment either directly or indirectly. Indirect action is through the secretion of cytokines and chemokines, which initiates a response from the surrounding cells [1]. Stem cells can also act directly by differentiating to a specific cell and hence contribute to tissue regeneration [2].

Mesenchymal stem cells derived from the bone marrow (BMSCs) have been commonly used in regenerative medicine, due to their capacity for in vivo differentiation into neurons, skeletal muscle progenitor cells, vascular endothelial cells, and cardiomyocytes [3,4]. However, bone marrow as a source of MSCs presents several limitations. The amount of marrow obtained from a patient is restricted due to donor site morbidity. Additionally, MSCs form a very small percentage, approximately <0.01%, of total nucleated cells in bone marrow, and the cells need to be expanded for 2–3 weeks in culture to obtain the required therapeutic dose. It may also be challenging to harvest bone marrow in smaller patients with smaller pelves [5,6,7].

Adipose tissue has recently gained attention as a source of mesenchymal stem cells (AdMSCs), because it is easier to acquire in larger volumes, the harvest is less painful, there are lower surgical risks, and there is minimal morbidity to the patient [8,9]. Adipose tissue can be collected from multiple sites, such as the inguinal, flank, omental, neck, scapular, falciform, and other regions [8,10]. Unlike liposuction, the fat is harvested from the body first and then minced. The minced tissue is then broken down by enzymatic digestion and centrifuged to obtain a pellet.

The pellet contains the stromal vascular fraction (SVF), which is composed of a mixture of cells including AdMSCs, erythrocytes, pericytes, fibroblasts, endothelial cells, smooth muscle cells, leukocytes, mast cells, and pre-adipocytes [11,12,13,14]. Most importantly, the number of MSCs in adipose tissue is higher than that in bone marrow [15]. One gram of adipose tissue yields approximately 5000 AdMSCs, while the yield of MSCs from bone marrow is 100–1000 cells per mL [16].

MSCs can be used in the treatment of various musculoskeletal injuries [17]. In a study in equine tendon regeneration, adipose-derived mononuclear cells induced improved fibre linearity, uniformity, and crimp pattern and a recovery of the tendon architecture after implantation in an experimentally induced tendon lesion [18]. In regenerative veterinary medicine, AdMSCs have been employed in dogs with chronic osteoarthritis (OA) which is generally synonymous with degenerative joint disease (DJD). DJD is one of the most common causes of lameness in dogs and may account for 20% or more of all canine referrals [19]. Studies have shown that dogs suffering from DJD, treated with autologous AdMSCs, had significantly improved scores for lameness, pain, and range of motion compared to those of control animals [11,20]. Another field of application of AdMSCs in veterinary medicine is management of bone defects after trauma or after a surgical resection of bone tumours or bone cysts [21]. Bone grafts or expanded AdMSCs or BMSCs in combination with scaffold materials can induce an osteoinductive effect that encourages osteogenic differentiation of stem cells [22]. There is a growing demand for AdMSCs in regenerative medicine, and it is therefore important to establish the best source of fat tissue to generate stem cells.

The aim of this study was to investigate AdMSCs from the falciform and flank regions by quantifying the stromal vascular fractions (SVFs), colony forming units (CFU), aspect ratio of the cells, as well as the days needed to count the CFU. We also aimed to report surgical site complications after harvesting the fat from the two different anatomical regions and compare the quality of fat contingent on the age and weight of the of the patient. This could help in the determination of the minimum amount of fat required to obtain AdMSCs for treatment of degenerative joint disease.

## 2. Materials and Methods

### 2.1. Patient Recruitment and Imaging Evaluation

With the informed consent of owners, fat tissue was harvested from dogs diagnosed with different levels of DJD, from either their flank (21 dogs) or falciform region (17 dogs). Fat tissue was harvested with the aim of growing stromal cells to inject into joint(s) affected by DJD as part of a multimodal therapy. A complete clinical evaluation was performed including a routine haematology and serum biochemistry to ensure the dogs could be safely sedated for the surgical procedure.

The site of fat harvest was based on the surgeon’s preference, depending on ease of harvest and perceived patient morbidity post-operatively, and therefore only one type of fat was harvested from each patient. A clinical assessment was performed, showing different levels of DJD, present mostly in the elbow (11 unilateral elbow joints, 26 bilateral elbow joints, 4 coxo-femoral joints, 1 bilateral tarsal joint, second and fifth metacarpal joints, 3 stifle joints, 1 bilateral metacarpal joint, 1 shoulder joint, 3 tarsal joints, 1 bilateral coxo-femoral joints, 3 bilateral stifle joints). All patients suffered from DJD and stem cell injection had been recommended as part of their treatment protocol. We evaluated the fat sample harvested from each patient.

### 2.2. Fat Harvest and Culture of MSCs

Dogs were premedicated with an injection of either medetomidine 5–10 µg/kg body weight (sedator, Eurovet Animal Health, Bladel, The Netherlands) or acepromazine 5–10 µg/kg body weight (acp, Elanco, Liverpool, UK) intravenously followed by an injection of propofol 1–4 mg/kg body weight (propoflo Plus Zoetis, Leatherhead, UK) intravenously to effect, and were maintained with isoflurane (isoflo Zoetis, Leatherhead, UK) mixed with oxygen. MSCs were obtained from either subcutaneous (flank) or visceral (falciform) fat. The surgery was performed by multiple ECVS (European College of Veterinary Surgeons, Zürich, Switzerland) accredited surgeons.

For harvesting adipose tissue from the flank region, an incision of approximately 6 cm in length was made on either the right or on the left region of the flank, dependant on the surgeon’s preference. Approximately 3–30 g of subcutaneous fat was collected by gentle blunt dissection while haemostatis was maintained. To obtain adipose tissue from the falciform region, a standard ventral midline incision of no longer than 10 cm was made, followed by gentle dissection of subcutaneous tissue and an incision in the cranial aspect of the linea alba. Approximately 3–30 g of adipose falciform fat were collected. In all cases, closure was per routine at the surgeon’s discretion.

Postoperative analgesia of the dogs was provided by the use of either methadone 0.2 mg/kg IV every 4–6 h (comfortan, Eurovet Animal Health, Bladel, The Netherlands) or buprenorphine 0.02 mg/kg IV (vetergesic, Ceva Labiana, Barcelona, Spain) every 6–8 h at the surgeon’s preference and after the assessment of the Glasgow pain score. Dogs were sent home the same day of the surgery. Some of the dogs were already on a non-steroidal anti-inflammatory drug chosen by their primary care veterinarian. There were no postoperative complications in either of the groups. The skin sutures were removed 10–14 days post-surgery and the wound in all cases was healed with no complications.

The adipose tissue was transferred directly in a sterile sample tube containing DMEM and 1% penicillin/streptomycin (P/S) (Sigma Aldrich, Darmstadt, Germany). The adipose tissue was processed by washing it with phosphate buffered saline (PBS) (Sigma Aldrich, Darmstadt, Germany) containing 1% P/S (Sigma Aldrich, Darmstadt, Germany), weighed using digital scales (Sartoris Entris, Göttingen, Germany), and then broken down using a scalpel. The minced adipose tissue was then digested on an orbital shaker at 140 rpm using 0.1% collagenase type I (Sigma Aldrich, Darmstadt, Germany) at 37 °C for 1.5 h. In total, 300 μL of 0.1% collagenase were used per gram of fat. Thereafter, the fat mixture was centrifuged at 644× g for 5 min to obtain a cell pellet [3,23].

### 2.3. Stromal Vascular Fraction (SVF) and Colony Forming Units (CFU)

The cell pellet (SVF) obtained from the centrifuged fat mixture was then re-suspended in growth media composed of DMEM supplemented with 20% (*v*/*v*) foetal calf serum (FCS) (First Link, Birmingham, UK) and 1% P/S solution. Then, 20 μL of this SVF solution was stained with trypan blue solution to count the total number of nucleated cells using a haemocytometer. The rest of the SVF was cultured in three T225 flasks (total area of 675 cm^2^) in growth media composed of Dulbecco’s Modified Eagle’s Medium (DMEM) with 20% foetal calf serum (FCS) and 1% penicillin and streptomycin (P/S) at 37 °C and 5% CO2. Approximately 3–7 days later, the growth of the cells was assessed and colony forming units (CFU) were counted. The growth media was changed after 3–7 days from initial plating to remove non-adherent cells and thereafter every 3–4 days.

Mesenchymal stromal stem cells must be plastic-adherent when maintained in standard culture conditions, and they are generally referred to as fibroblastoid colony forming cells, with an ability to generate colonies [6,24,25]. Therefore, each colony originates from a single stromal cell. Colonies were counted at ×5 magnification under a phase-contrast light microscope (Leica DMIL LED Fluo, Wetzlar, Germany). The CFU were counted by dividing the flask into twenty-five grid regions (3 cm by 3 cm), and counting CFU in six randomly selected regions, and then calculating the average number of CFU per flask [26]. When cells had grown to 80–90% confluency, the cells were passaged or cryopreserved for future OA injections.

Additionally, to establish whether there was the minimum amount of fat needed from either the flank or falciform regions for treatment therapy, we also compared the SVFs and CFU for different weights of fat from the same patients. The harvested fat sample was divided into two different weights, processed as described above to count the SVFs, and cultured separately for CFU count.

### 2.4. Cell Morphology

MSCs exhibit a fibroblastic or spindle-like shape, and it is therefore important to measure their length-to-width ratio [24,27]. The cells at passage 1 were imaged using light microscopy (Leica, Wetzlar, Germany) at 10× magnification. Aspect ratio (AR) is an important measurement of cell shape [28]. The length and width of cells from fat derived from both the falciform and the flank regions (n = 5 each) were measured using Image J software (LOCI, University of Wisconsin, Madison, WI, USA) to calculate the aspect ratio by dividing the length of cells by the width.

### 2.5. Statistical Analysis

The data in this study were assessed for normal distribution using a Kolmogorov–Smirnov test. A Pearson’s correlation test was used to determine the correlation between the data. All data were statistically evaluated using SPSS statistical software 25 (Chicago, IL, USA). If the data were parametric (normally distributed), the results were presented as mean ± standard deviation (SD) and statistical comparison was carried out using an independent sample t test. If the data were non-parametric, the results were presented as median (range) and analysed using a Mann–Whitney U test. All *p* values of <0.05 were considered to be statistically significant.

## 3. Results

### 3.1. Patient Recruitment and Imaging Evaluation

Harvest of adipose tissue and culture of AdMSCs was successful in all dogs, with no post-operative complications. Breeds included were small to large dogs (2 Border Collies, 1 Boxer, 11 Labradors, 1 Rottweiler, 4 German Shepherd dogs, 1 English Springer Spaniel, 2 Jack Russell Terriers, 3 Cross breeds, 2 Staffordshire terriers, 1 Russian terrier, 1 Bulldog, 1 Chow Chow, 1 Crossed English Mastiff, 1 West Highland White Terrier, 1 Pug, 2 Golden Retrievers, 1 Pyrenean Mountain dog).

Table 1 shows that the average weight of fat harvested from the flank was 8.4 ± 3.7 g and from the falciform was 10.1 ± 6.6 g. It took on average 3.8 days and 4.1 days for CFU to be obtained from each site, respectively. In both groups, if the weight of the adipose tissue was more than 10 g, it was divided into two parts.

### 3.2. Fat Harvest and Culture of MSCs

Using the Mann–Whitney U test, we found no significant difference between both sites with respect to the number of days needed to count the CFU (*p* = 0.578). The median and the range for both groups was 3 ± 2–10.

**Figure 1:**
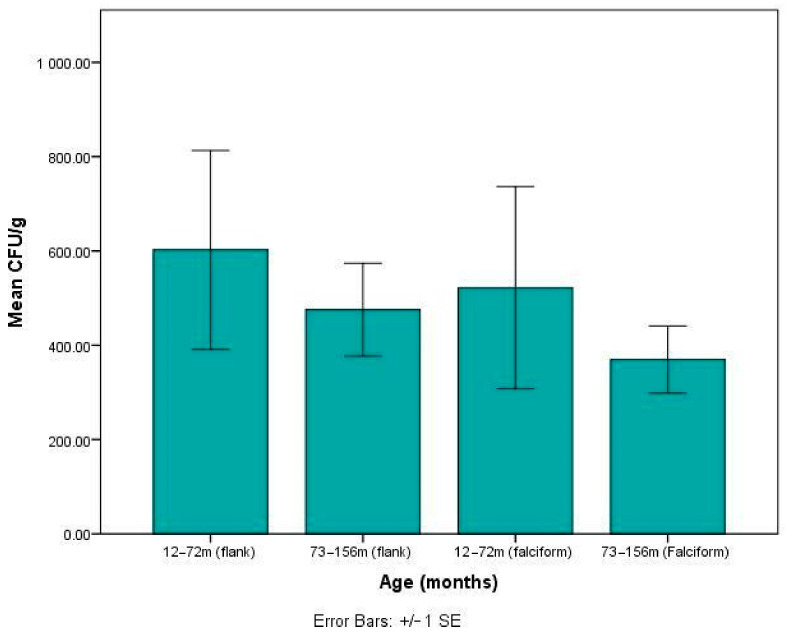
The colony forming units (CFU)/g of fat in relationship to the age of the patient showing no correlation in either of the anatomical region.

Age vs. CFU/g: Dogs from each group were divided into subgroups of young (12–72 months) and old (73–156 months). Ten dogs in the flank group and only 3 dogs in the falciform group met the criteria of being aged between 12–72 months. Seven dogs in the flank group and 10 in the falciform group met the criteria of being aged between 73–156 months. This relationship was analysed using a Mann–Whitney U test. There was no significant difference in CFU/g between the young and old patients for fat harvested from either the flank (*p* = 0.618) or falciform (*p* = 0.576) region. Additionally, irrespective of age, the number of stem cells (CFU) per gram of fat was not significantly different between falciform and flank groups (Figure 1).

**Figure 2:**
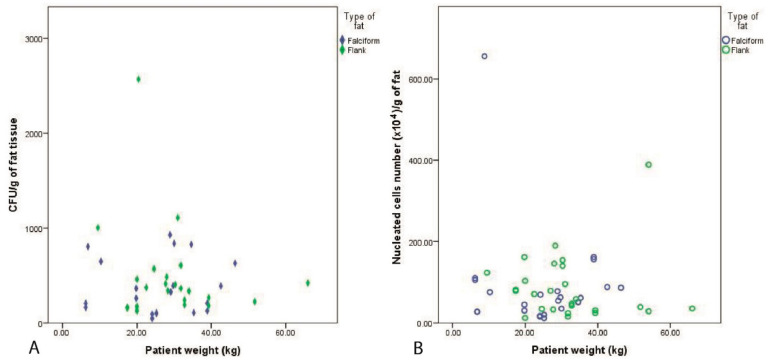
Pearson’s correlation demonstrating no significant difference in variability of nucleated cells and CFU/g of cells vs. weight of the patient in the fat harvested from the falciform and flank regions. Weight of the patient vs. CFU/g for the fat harvested from the falciform (*p* = 0.890) and flank regions (*p* = 0.333) (**A**). The weight of the patient vs. nucleated cells in stromal vascular fractions (SVFs) per gram of fat was not significantly different between the two harvest sites: flank (*p*= 0.874) and falciform (*p* = 0.966) (**B**).

Patient weight vs. CFU/g and SVF/g of fat: Pearson’s correlation showed that there was no significant difference between the weight of the patient and the number of stem cells present in the CFU/g for the fat harvested from the falciform (r = 0.033, *p* = 0.890) and flank groups (r = −0.211, *p* = 0.333). The nucleated cells (SVF per gram of fat), obtained was also not dependant on the patient weight and was not significantly different between the two harvest sites: flank (r = 0.033, *p* = 0.874) or falciform (r = −0.009, *p* = 0.966) (Figure 2).

**Figure 3:**
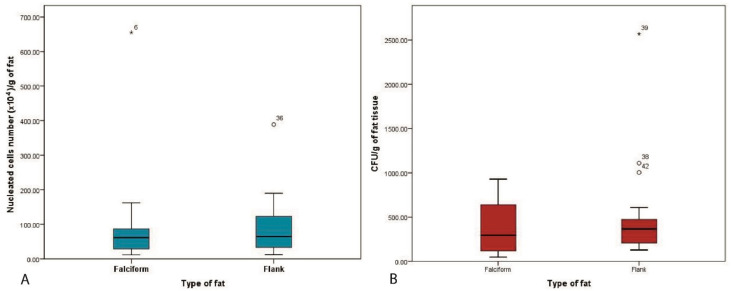
The SVFs and CFU per gram of fat from the two anatomical regions. There was no significant difference in the number of nucleated cells (*p* = 0.602) (**A**) or CFU/g of fat (*p* = 0.312) (**B**) harvested from the falciform or the flank region.

Number of endothelial cells or MSCs (CFU) per nucleated cells in the SVF: We calculated that for the fat harvested from the flank, there was 1 endothelial or stem cell for every 1145 (±1344) cells in the SVF and for the fat harvested from the falciform, there was 1 endothelial or stem cell for every 1247 (±1570) cells. These results are quite similar, and a Mann–Whitney U test demonstrated no significant difference in the number of nucleated cells in fat obtained from the two sites (*p* = 0.602) (Figure 3).

**Figure 4:**
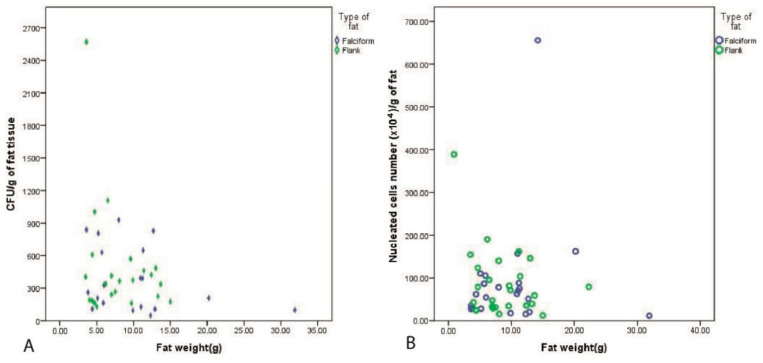
A positive Pearson’s correlation was observed between the harvested weight of the fat and quantified CFU/g for the fat from the flank (r = 0.478, *p* = 0.021), but this was not reported for the falciform fat. (**A**) Additionally, no correlation was recorded with the fat weight and nucleated cell number (SVF/g) (**B**).

Weight of the harvested fat vs. CFU and SVF: Pearson’s correlation demonstrated a significant positive relationship between the weight of the fat obtained from the flank and CFU/g (r = 0.478, *p* = 0.021), but this was not observed for the falciform fat (r = −0.204, *p* = 0.417). No significant correlation was observed between the nucleated cells in SVFs (SVF/g) and the weight of the fat harvested from the flank (r = 0.36, *p* = 0.537) and the falciform (r = −0.204, *p* = 0.376) (Figure 4).

**Figure 5:**
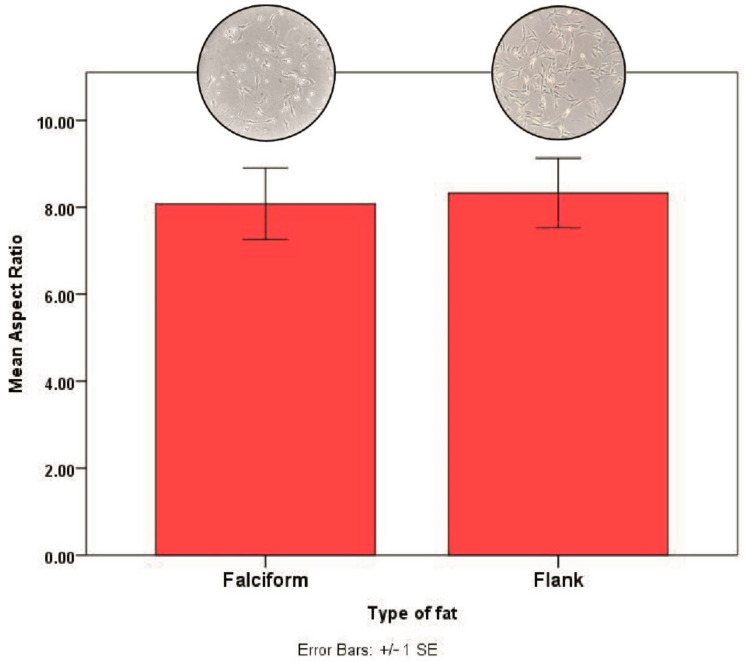
The mean aspect ratio (ratio of length/width) of AdMSCs harvested from the flank and falciform region were very similar (*p* = 0.83). The error bars represent ± 1 standard error. Amplification factor of the image-5x.

Cell morphology: AdMSCs obtained from falciform or flank regions have spindle-like features. The mean aspect ratio for AdMSCs from the flank region was 8.33 ± 1.96 and from the falciform region was 8.08 ± 2.02, and this was not significantly different (*p* = 0.83) when analysed using an independent samples *t* test (Figure 5).

## 4. Discussion

In this in vitro study, we explored the quality of the fat harvested from the flank and the falciform regions of canine patients by quantifying the SVFs and CFU per gram of fat harvested, and by comparing this with the age and weight of each patient. This study was conducted under clinical conditions with client-owned dogs suffering from different degrees of DJD, present mostly in the elbow joint, for which stem cell regenerative therapy was part of their prescribed medical management protocol. We chose to harvest AdMSCs due to their ease of tissue collection, high initial cell yields, and robust in vitro proliferative capacity [29,30]. Irrespective of the harvest site, the stem cell quantity, measured by the CFU counts, was not significantly different. Additionally, there was no difference in cell morphology between the adipose tissue harvested from the two anatomical regions: flank and falciform.

Various anatomical sites in the body can be sources of stem cells. For example, the synovium has been shown to generate cells that have a higher proliferation and chondrogenic differentiation compared to MSCs from adipose tissue and bone marrow [31]. In rabbits, however, it has been shown that synovium and bone-marrow MSCs had greater in vivo chondrogenic potential than did adipose and muscle MSCs [32]. In a study of twenty clinically healthy dogs investigating canine adipose tissue cultured from subcutaneous and visceral regions, no significant difference was found in terms of cell yield [2]. These results are similar to our study, but our case population is more clinically relevant, constituting different breeds with multiple joint DJD and a wide age and bodyweight range, where the yield of cells could be more variable.

There are several studies in horses and humans that have shown differences in AdMSCs harvested from various regions of the body in terms of cell viability, proliferation, and differentiation [33,34,35,36,37,38]. It has been found in dogs that adipose collection from the thoracic wall and the inguinal region may yield more viable cells per gram than the falciform region [8]. The ideal site for collecting adipose tissue from a clinical perspective would be the site that causes minimal morbidity, consistently has a large amount of fat for extraction, and yields a large SVF, which should be proportional to the fat obtained [8]. Moreover, when selecting an adipose harvest location for intra-articular therapy of DJD, the surgeon must consider patient factors, biologic factors, and his or her own comfort level with the procedure [8]. In this study, no complications were observed in any of the harvest sites; therefore, adipose tissue from both the falciform and flank regions are equally viable and indicated for fat harvest to yield stem cells.

Age and weight of the patient has been shown to affect the quality of stem cells harvested from clinical and experimental animals [8,39]. Additionally, human BMSCs from younger patients have been shown to have better differentiation and proliferation properties compared to BMSCs from older patients [40]. In dogs, it was found that younger dogs might have a higher median viable cells per gram [8]. In this study, age of the patient did not produce any significant difference for the CFU/g of the adipose sample. This could possibly be related to our small number of patients (type 2 error) although using non-parametric analysis we also showed no significant differences between age groups. Our results demonstrate that stem cells can be obtained from the adipose tissue of any dog, of any weight, irrespective of their maturity, which indicates a broad remit for stem cell therapy of DJD in clinical canine patients. In humans, some studies intimate a tendency for higher cumulative population doublings in MSCs from younger donors [41]. However, in another human study, no significant correlation was detected between the frequency of ASCs and the age of the donor [12]. Guercio found no difference in terms of yield of cells between young and adult dogs [42].

We also investigated the weight of the fat with respect to the number of CFU/g of fat and found that the number of stromal cells from the flank region was positively correlated with the weight of the fat (r = 0.478 for the CFU/g of fat and 0.136 for the SVF/g). Interestingly, fat from the falciform region yielded a negative correlation between the weight of the falciform fat versus the CFU/g and SVF/g, although this was statistically insignificant. This could potentially be attributable to the macroscopic structure of falciform fat and the difficulty of mincing and enzymatically digesting a large fat sample. We standardized the time for enzymatic digestion to 1.5 h for all fat samples. When comparing to other studies for adipose tissue collection, the caudal scapular subcutaneous tissue region required more dissection and did not yield as much adipose tissue as did the falciform (30 g) fat region. In that study also, patient body weight and body condition score were not significantly associated with total cell concentration [43]. In humans, adipose tissue is readily abundant and more accessible than human bone marrow [44].

We therefore propose both the flank and falciform as adequate regions for harvesting MSCs with no postoperative complications or rate of morbidity and without the necessity of harvesting a large amount of fat.

This study has some limitations with respect to the variability of the breeds, which we could not control since it was a clinical study. It would be interesting to investigate the CD markers, tri-differentiation, and proliferation capability of these cells in vitro. We understand we cultured a mixed population of stromal cells, so future studies could use a cell sorter to obtain a pure population of MSCs from the SVF. Clearly the number of cases was contingent on how long the study ran for, and perhaps greater statistical significance may be established with larger case numbers. However, a larger number of dogs would not have been expected to yield different results as we showed few differences with the low number of individuals investigated.

## 5. Conclusions

Harvesting fat for stem cell therapy for intra-articular therapy of DJD can be an easy and fast process when obtaining it either from the flank or the falciform region, and it is not age- or weight-dependent. Both sites showed 0% postoperative complications and based on number of stem cells harvested per gram of fat, the harvest site for clinical canine patients can be left to the surgeon’s discretion and comfort.

## Figures and Tables

**Table 1:** Population characteristics such as harvest site, number of dogs, median (range) age, mean ± SD body weight of dogs, and mean ± SD weight of fat from the flank and falciform regions.

Harvest Site	Flank	Falciform
Dog numbers	21	17
Median age (months)	66	114.5
Mean patient body weight (kg)	29.4 ± 12.0	26.1 ± 11.9

## Data Availability

The data presented in this study are available on request from the corresponding author. The authors hold an electronic file of the patient data included in this study. Personal data about this patient is anonymised when used in any form of publications and presentations. All personal data about every dog is kept strictly confidential and does not leave the hospital.
